# Early hyperbaric oxygen therapy is associated with favorable outcome in patients with iatrogenic cerebral arterial gas embolism: systematic review and individual patient data meta-analysis of observational studies

**DOI:** 10.1186/s13054-023-04563-x

**Published:** 2023-07-12

**Authors:** Raoul A. Fakkert, Noa Karlas, Patrick Schober, Nina C. Weber, Benedikt Preckel, Robert A. van Hulst, Robert P. Weenink

**Affiliations:** 1 Department of Anesthesiology, Amsterdam UMC Location University of Amsterdam, Meibergdreef 9, 1105 AZ Amsterdam, The Netherlands; 2 Department of Anesthesiology, Amsterdam UMC Location Vrije Universiteit Amsterdam, De Boelelaan 1117, Amsterdam, The Netherlands; 3 Hyperbaric Medicine, Amsterdam UMC Location University of Amsterdam, Meibergdreef 9, Amsterdam, The Netherlands; 4 Laboratory of Experimental Intensive Care and Anesthesiology, Amsterdam UMC Location University of Amsterdam, Meibergdreef 9, Amsterdam, The Netherlands

**Keywords:** Air embolism, Hyperbaric oxygenation, Iatrogenic disease, Embolic stroke

## Abstract

**Background:**

Iatrogenic cerebral arterial gas embolism (CAGE) caused by invasive medical procedures may be treated with hyperbaric oxygen therapy (HBOT). Previous studies suggested that initiation of HBOT within 6–8 h is associated with higher probability of favorable outcome, when compared to time-to-HBOT beyond 8 h. We performed a group level and individual patient level meta-analysis of observational studies, to evaluate the relationship between time-to-HBOT and outcome after iatrogenic CAGE.

**Methods:**

We systematically searched for studies reporting on time-to-HBOT and outcome in patients with iatrogenic CAGE. On group level, we meta-analyzed the differences between median time-to-HBOT in patients with favorable versus unfavorable outcome. On individual patient level, we analyzed the relationship between time-to-HBOT and probability of favorable outcome in a generalized linear mixed effects model.

**Results:**

Group level meta-analysis (ten studies, 263 patients) shows that patients with favorable outcome were treated with HBOT 2.4 h (95% CI 0.6–9.7) earlier than patients with unfavorable outcome. The generalized linear mixed effects model (eight studies, 126 patients) shows a significant relationship between time-to-HBOT and probability of favorable outcome (*p* = 0.013) that remains significant after correcting for severity of manifestations (*p* = 0.041). Probability of favorable outcome decreases from approximately 65% when HBOT is started immediately, to 30% when HBOT is delayed for 15 h.

**Conclusions:**

Increased time-to-HBOT is associated with decreased probability of favorable outcome in iatrogenic CAGE. This suggests that early initiation of HBOT in iatrogenic CAGE is of vital importance.

**Supplementary Information:**

The online version contains supplementary material available at 10.1186/s13054-023-04563-x.

## Introduction

Invasive medical procedures can lead to accidental entrainment of gas into the circulation. Vascular gas entry can complicate virtually any procedure, but typical categories include cardiac surgery, lung biopsy, interventional radiology and procedures involving central venous catheters [1]. When gas bubbles flow to the cerebral circulation, they cause cerebral arterial gas embolism (CAGE), which manifests as stroke [2]. Other than supportive therapy and increasing the fraction of inspired oxygen, the only established treatment for CAGE is hyperbaric oxygen therapy (HBOT), which decreases the size of bubbles because of increased ambient pressure and rapid denitrogenation, and optimizes oxygenation of marginally perfused brain tissue [3].

Apart from iatrogenic causes, CAGE can also occur in divers breathing compressed gas (e.g. via self-contained underwater breathing apparatus), who may develop pulmonary barotrauma during ascent. In these cases, air from ruptured alveoli enters the pulmonary veins and ultimately may lodge into the cerebral arteries [4]. Since CAGE is a well-known complication of diving and submarine escape exercises, clinical awareness is high, and during certain high-risk activities HBOT may be immediately available [5]. The excellent results obtained in cases where HBOT is rapidly initiated [5] has led to the class I recommendation for HBOT in the treatment of CAGE [2, 6], despite the fact that no randomized trial has ever been performed on this subject. Indeed, many experts would regard a trial in which the control group does not receive HBOT as unethical [7]. Nevertheless, since the recommendation for HBOT in CAGE is currently based primarily on plausible rationale, animal research and cohort studies, many clinicians remain reluctant to refer patients for HBOT, leading to underuse of a possibly highly effective intervention. This is compounded by the suboptimal availability of recompression chambers that provide emergency HBOT service [8].

Given the lack of randomized studies into HBOT for CAGE, other methods to judge its efficacy should be pursued. One possibility might be to determine the relationship between delay until start of HBOT and outcome, the rationale being that if earlier initiation of HBOT leads to better outcome, this is strong evidence that HBOT is indeed an effective therapy. In the present study we systematically searched for all studies on iatrogenic CAGE that reported time-to-HBOT and outcome, and aimed to aggregate data to answer the question whether earlier initiation of HBOT is associated with favorable outcome.

## Methods

### Search and study selection

The protocol for this systematic review and meta-analysis was published in PROSPERO (identification number CRD42022362516). With the help of a clinical librarian, Medline and Embase databases were searched on October 11th, 2022. The searches combined terms for ‘iatrogenic disease’, ‘brain’, ‘gas embolism’ and ‘hyperbaric oxygen therapy’, the full search protocols can be found in the Additional file 1. Inclusion of articles was done in two rounds (first based on title and abstract, subsequently based on full text) by two authors (RPW and NK) independently. Disagreement between the two reviewers was solved by discussion. Inclusion was based on the following criteria: (1) study is available in English; (2) study reports on a cohort of patients with iatrogenic CAGE included in a defined period of time (i.e. case reports and case series were excluded); (3) study reports both time-to-HBOT and clinical outcome, either on group level or individual patient level. All methods to describe clinical outcome were accepted, for instance ‘favorable’ vs ‘unfavorable’, outcome scores such as Glasgow Outcome Scale, or qualitative description of neurological outcome. Additional studies were identified by checking the references of the included articles. For studies that might be of relevance, but in their published form contained too little information to be used for meta-analysis, we contacted the authors to obtain additional information needed for final inclusion. This was also done for studies that reported only group level data; in these cases the authors were contacted for individual patient data. Quality of studies was assessed using the Newcastle–Ottawa scale for cohort studies.

### Data extraction

From the included studies we extracted the following variables: age, sex, causative medical procedure, presenting symptoms, time between occurrence of CAGE and start of HBOT, clinical outcome, and timepoint at which outcome was determined. In studies that reported group data for ‘favorable’ vs. ‘unfavorable’ outcome we noted the criteria for these classifications. For studies that reported individual patient data, we classified all patients as having either ‘favorable’ or ‘unfavorable’ outcome, based on the following criteria: favorable outcome was scored when outcome was described as ‘total recovery’, ‘complete response’, ‘complete recovery’, ‘excellent recovery’, ‘recovery’, extended Glasgow Outcome Scale score of 7–8, modified Rankin Scale score of 0–1, or described in such terms that resumption of normal life with only minor neurological and/or psychological deficits was apparent. In studies that reported individual patient data, we classified symptoms of each patient in the following categories of increasing severity: ‘nonspecific symptoms’, ‘focal neurological symptoms’, ‘altered consciousness’, ‘coma’ and ‘circulatory arrest’. If patients could be categorized in multiple categories, they were classified into the highest category. For instance, a patient who exhibited focal neurological symptoms but later developed coma, would be scored in the category ‘coma’. Scoring of both symptoms and outcome was done independently by two authors (RPW and NK) and any discrepancies were solved by discussion.

### Statistics and meta-analysis

Data were analyzed using Stata 17.0 (StataCorp, College Station, TX) and R version 4.2.2 (R Foundation for Statistical Computing, Vienna, Austria). The difference in the median delay to treatment between the two outcome groups was meta-analyzed across studies (individual patient studies as well as studies reporting group data) using the median of the differences of medians method as described by McGrath et al. [9], using the ‘metamedian’ package in R. The pooled estimate of the difference of medians as well as its 95% confidence interval are reported.

On individual patient level, we analyzed the association between the delay to treatment and the dichotomized outcome (i.e., favorable vs. unfavorable outcome) with a generalized linear mixed effects model with a logit link function and a random intercept for study [10]. Because circulatory arrest is inherently associated with unfavorable outcomes and turned out to be a perfect predictor for an unfavorable outcome in our dataset, patients with circulatory arrest were excluded from the patient level data analysis. To allow for a nonlinear association between the delay to treatment and the logit of the outcome, the delay to treatment was modeled as a restricted cubic spline with three knots, with the number of knots being based on Bayesian Information Criteria. Patient symptoms were identified as a potential key confounder based on theoretical considerations, e.g. more severe symptoms may lead to earlier treatment but also to worse outcomes. In order to adjust for severity of manifestations while accounting for missing data on this variable, multiple imputation of 100 data sets was performed using chained equations, and the adjusted relationship between the delay to treatment and the outcome was computed across imputed datasets using Rubin’s rules [11]. A two-sided *p* value < 0.05 was considered statistically significant.

## Results

**Fig. 1:**
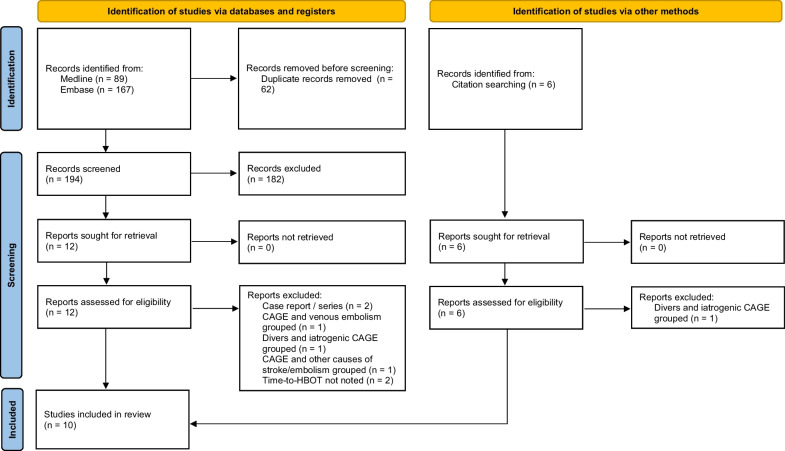
PRISMA diagram

The PRISMA flow diagram of the search and selection process (Fig. 1, with additional information and quality assessment in the Additional file 1) shows that a total of 10 studies were used in the analysis. Of these 10 studies, eight contained individual patient level data, and two reported only group level data. The studies are summarized in Table 1, with additional information in the Additional file 1. The definition of favorable outcome as used in those two studies reporting group level data was consistent with our own definition of favorable outcome as used for scoring of the individual patient data, therefore we were able to combine all studies for meta-analysis.

**Table 1:** Summary of included studies

Author	Number of patients	Age	% female	Favorable outcome definition	% favorable outcome	Time-to-HBOT in patients with favorable outcome (median, IQR)	Time-to-HBOT in patients with unfavorable outcome (median, IQR)
*Studies with only group data*							
Beevor and Frawley [12]	45	Mean 56 years (SD 22)	44%	GOSE 7–8	60%	8.8 (8.1) h	16.5 (15.1) h
Blanc et al. [13]	86	Mean 52 years (SD 19)	36%	Total recovery from all neurological deficits	58%	3 (3) h	4.75 (5.25) h
*Studies with individual patient data*							
Benson et al. [14]	19	Mean 45 years (SD 21)	63%	Derived by us based on qualitative description by authors	53%	6.00 (19.9) h	5.50 (4.19) h
Kol et al. [15]	6	Median 50 years (IQR 23)	50%	Complete recovery	33%	2.50 (n/a) h	19.0 (2.75) h
Massey et al. [16]	14	Mean 48 years (SD 21)	50%	Total resolution	7.1%	n/a	12.0 (22.8) h
Muller et al. [17]	15 (10 used, 4 unknown delay, 1 no hyperbaric oxygen)	Mean 62 years (SD 20)	73%	mRS 0–1	50%	5.17 (6.22) h	8.21 (12.2) h
Murphy et al. [18]	16 (14 used, 2 unknown delay)	Median 61 (IQR 23)	14%	Complete response	50%	3.00 (11.0) h	11.0 (9.00) h
Takahashi et al. [19]	34 (18 used, 16 non-cerebral gas emboli)	Mean 45 years (SD 17)	33%	Excellent	44%	22.5 (21.5) h	23.3 (20.6) h
Tekle et al. [20]	36 (34 used, in 2 patients outcome could not be classified)	Mean 48 (SD 21)	56%	Derived by us based on qualitative description by authors	29%	5.50 (6.74) h	6.38 (8.67) h
Ziser et al. [21]	17	Median 62 (SD 22)	41%	Recovery	47%	3.00 (2.45) h	17.0 (12.8) h

*IQR* interquartile range, *SD* standard deviation, *GOSE* extended Glasgow Outcome Scale, *mRS* modified Rankin Scale. Age is reported as mean and SD when normally distributed, otherwise as median and IQR

**Fig. 2:**
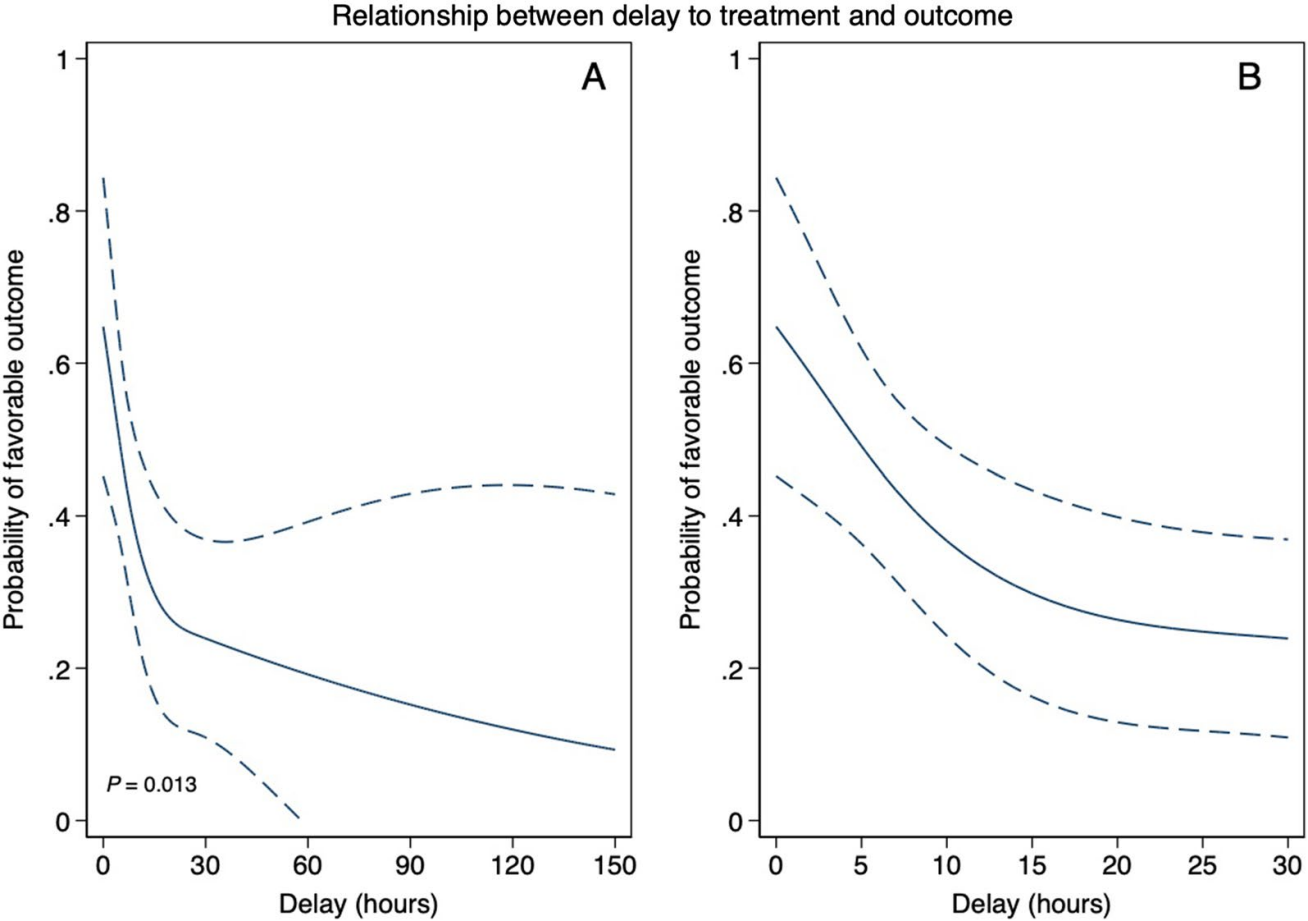
Graphical results of the generalized linear model, with probability of favorable outcome plotted in relation to time-to-HBOT. Dashed lines are 95% confidence intervals. The left-hand figure **A** displays the data with the delay plotted up to 150 h, whereas the figure on the right **B** shows the same data but zoomed in to a timeframe of 30 h, in order to show the first hours in more detail

In the meta-analysis of group level data (ten studies, 263 patients), patients with favorable outcome were treated significantly earlier than those with unfavorable outcome (pooled difference in medians 2.4 h, 95% CI 0.6–9.7). On patient level data (eight studies, 126 patients), the generalized linear mixed effects model shows a significant relationship between time-to-HBOT and outcome (*p* = 0.013). The output of the model is presented in Fig. 2 and shows a probability of favorable outcome of approximately 65% when HBOT is started immediately, which declines to approximately 30% when HBOT is started after 15 h. After approximately 20–25 h, additional delay does not seem to have an evident relationship with outcome. After multiple imputation and adjustment for severity of manifestations, the significant relationship persisted (*p* = 0.041).

## Discussion

We have shown that in patients with favorable outcome after CAGE, median time-to-HBOT was approximately 2.4 h shorter than in patients with unfavorable outcome. Individual patient data shows a relationship between time-to-HBOT and probability of favorable outcome, which decreases from approximately 65% when HBOT is started immediately, to 30% when HBOT is started after approximately 15 h. Our results partly align with the conclusions drawn in several of the studies included in this meta-analysis, as well as a large prospective cohort study [22], that HBOT seems to be most effective when initiated no later than 6–8 h after onset of symptoms. Our data extend the conclusions from these studies, and suggest that the relationship between time-to-HBOT and outcome should not be seen as an on–off phenomenon with a cutoff point at 6–8 h, but rather as a continuum in which sooner is better.

We believe the association between early HBOT and favorable outcome in CAGE is highly suggestive of an actual therapeutic effect of HBOT, and that our results provide compelling evidence to refer all cases of iatrogenic CAGE to a hyperbaric facility at the earliest opportunity. An association, however, does not prove causality. Although we have adjusted our model for severity of manifestations, residual confounding may still be present. For instance, delayed start of HBOT may be indicative of delayed recognition and diagnosis of CAGE, in which cases not only HBOT is delayed, but possibly also other important aspects of supportive management, such as application of normobaric hyperoxia. It may be possible that patients with early start of HBOT were already admitted to a center with a hyperbaric chamber, and the presence of such a chamber may be related to increased clinical awareness and improved treatment of conditions that require HBOT, such as CAGE.

Apart from iatrogenic causes, CAGE can also occur in pulmonary barotrauma. During activities in which subjects have a high risk of pulmonary barotrauma, such as submarine escape training, clinical suspicion for CAGE is high and recompression therapy may be immediately available. Brooks et al. [5] have demonstrated that under these circumstances, a recovery percentage of 91% may be attained, and another study noted a curation rate of 74% in divers with CAGE who were treated with HBOT within 2 h [23]. The fact that in our data the success rate with immediate initiation of HBOT was only 65% may possibly be accounted for by the obvious differences between healthy submarine escape trainees and divers, and clinical patients (with potential co-morbidities) who suffer CAGE due to an invasive medical procedure.

In our data, after 20–25 h the influence of additional delay on outcome is much less clear than in the first hours. Although this result should be regarded with caution, because of increasingly lower numbers of subjects (and correspondingly wider confidence intervals) with longer delays, it may indicate that after this time recovery is mostly determined by the natural course of the disease, and the value of HBOT is limited. This would suggest that in cases in which HBOT cannot be started within this timeframe, because of delay in recognition and/or transportation time to a hyperbaric facility, one could consider to withhold HBOT and focus on optimal supportive therapy, including normobaric oxygen. However, as several cases have been published with delays of more than 24 h, in which there was a clear temporal relationship between HBOT and improvement of neurological status [24–26], we recommend to consider HBOT even in cases with prolonged delay.

Strengths of our study are the systematic literature search, our efforts to obtain additional information from the original authors, classification of symptoms and outcome by two independent reviewers, adjustment of our model for severity of manifestations, and the combination of individual patient level and group level data to analyze our results. Limitations are the fact that despite our efforts we received only one reply to our requests for additional data, and therefore our conclusions are based on less patients than would otherwise have been possible. Also, we were only able to categorize patients into favorable versus unfavorable outcome, where a more refined distinction into several outcome categories would have provided more insight. Furthermore, the included studies used various timepoints for determination of clinical outcome, which introduced some heterogeneity. Additionally, we had to exclude patients in circulatory arrest from the patient level data analysis, because the fact that it was a perfect predictor for unfavorable precluded its use in the generalized linear mixed effects model. It should be stressed that this does not infer that a patient who experienced a period of circulatory arrest should not be considered for HBOT. The number of patients in arrest in our dataset was very small (*n* = 6) and it would be unwise to extrapolate the unfavorable outcome in these six patients to the entire iatrogenic CAGE population. A last limitation of our study is that we did not derive data on type and number of HBOT sessions. We believe that future research into iatrogenic CAGE should focus on high-quality observational studies, and possibly randomized trials that investigate various HBOT regimens, such as single versus repeated HBOT sessions.

In conclusion, we have shown that earlier start of HBOT is associated with an increased probability of favorable outcome in patients with iatrogenic CAGE, and that the probability of favorable outcome decreases from approximately 65% with immediate initiation of HBOT, to 30% when HBOT is delayed for 15 h. This calls for early recognition of iatrogenic CAGE and expeditious initiation of HBOT.

## Supplementary Information

**Additional file 1**. Search strategy, protocol, quality assessment of included studies, and additional study information.

## Data Availability

The datasets used and/or analyzed during the current study are available from the corresponding author on reasonable request.

## Abbreviations

CAGE: Cerebral arterial gas embolism
HBOT: Hyperbaric oxygen therapy
