# Supplementary material for: Early hyperbaric oxygen therapy is associated with favorable outcome in patients with iatrogenic cerebral arterial gas embolism: systematic review and individual patient data meta-analysis of observational studies
Source: Crit Care. 2023 Jul 12;27:282. doi: 10.1186/s13054-023-04563-x (PMC10337083; doi:10.1186/s13054-023-04563-x)
Supplement: Supplementary file 1 — Additional file 1. Search strategy, protocol, quality assessment of included studies, and additional study information. [file 13054_2023_4563_MOESM1_ESM.docx]

**Supplemental material**

1. Search strategy

Search performed 11 OCT 22 🡪 256 results

Deduplicated 11 OCT 22 🡪 194 results

Title and abstract screening 12 OCT 22 🡪 12 to full text screening

Snowballing completed 01 NOV 22 🡪 6 added 🡪 18 total

- 2 studies were excluded because they were case reports / case series
- 6 studies were excluded because they could only be used when individual patient level data was available; authors were contacted if contact information was available, but no response was received

Final included articles 🡪 10 total

- 7 studies that reported individual patient level data in the original article
- 1 study from which we received individual patient level data from the authors
- 2 studies that only reported group level data; authors were contacted for group level data, but no response was received

1. Search protocols for Medline and Embase

**Medline**

| **#** | **Searches** |
| --- | --- |
| 1 | exp Iatrogenic Disease/ |
| 2 | exp Medical Errors/ or exp Diagnostic Errors/ |
| 3 | Patient Harm/ |
| 4 | (iatrogenic* or error* or "hospital acquired condition*" or harm* or acciden*).ti,ab,kf. |
| 5 | 1 or 2 or 3 or 4 |
| 6 | Embolism, Air/ |
| 7 | ((air adj3 embol*) or (gas adj3 embol*)).ti,ab,kf. |
| 8 | 6 or 7 |
| 9 | exp Central Nervous System/ |
| 10 | ("central nerv*" or cerebr* or CNS or "C.N.S." or brain*).ti,ab,kf. |
| 11 | 9 or 10 |
| 12 | exp Oxygen Inhalation Therapy/ |
| 13 | ("hyperbaric oxygen*" or HBO or HBO2 or HBOT or hyperbar* or "HBO-therap*" or "high tension oxygen*" or "high tension O2").ti,ab,kf. |
| 14 | 12 or 13 |
| 15 | 5 and 8 and 11 and 14 |

**Embase**

| **#** | **Searches** |
| --- | --- |
| 1 | exp iatrogenic disease/ |
| 2 | exp medical error/ |
| 3 | patient harm/ |
| 4 | (iatrogenic* or error* or "hospital acquired condition*" or harm* or acciden*).ti,ab,kf. |
| 5 | 1 or 2 or 3 or 4 |
| 6 | air embolism/ |
| 7 | ((air adj3 embol*) or (gas adj3 embol*)).ti,ab,kf. |
| 8 | 6 or 7 |
| 9 | exp central nervous system/ |
| 10 | ("central nerv*" or cerebr* or CNS or "C.N.S." or brain*).ti,ab,kf. |
| 11 | 9 or 10 |
| 12 | 8 and 11 |
| 13 | 5 or 12 |
| 14 | exp oxygen therapy/ |
| 15 | ("hyperbaric oxygen*" or HBO or HBO2 or HBOT or hyperbar* or "HBO-therap*" or "high tension oxygen*" or "high tension O2").ti,ab,kf. |
| 16 | 14 or 15 |
| 17 | 5 and 8 and 11 and 16 |

1. Assessment of study quality using Newcastle-Ottawa Quality Assessment Scale for cohort studies

| **Author** | **S1** | **S2** | **S3** | **S4** | **C1** | **O1** | **O2** | **O3** |
| --- | --- | --- | --- | --- | --- | --- | --- | --- |
| Beevor |  |  |  |  |  |  |  |  |
| Benson |  |  |  |  |  |  |  |  |
| Blanc |  |  |  |  |  |  |  |  |
| Kol | Note 1 |  |  |  |  | Note 2 | Note 3 |  |
| Massey |  |  |  |  |  |  | Note 3 |  |
| Muller |  |  |  |  |  |  |  |  |
| Murphy | Note 1 |  |  |  |  | Note 2 | Note 3 |  |
| Takahashi | Note 1 |  |  |  |  | Note 2 | Note 3 |  |
| Tekle |  |  |  |  |  |  | Note 4 |  |
| Ziser |  |  |  |  |  |  |  |  |

The abbreviations in the column headers refer to the eight questions of the Newcastle-Ottawa scale (see next page). No study includes a non-exposed cohort, i.e. a group of patients that did not experience cerebral gas embolism. Therefore, questions S2 and C1 cannot be answered, these are marked yellow.

Notes:

1. In these studies, it is not clear how the included patients were identified. The text merely mentions a period of inclusion, but not how patients were identified (e.g. through checking of medical records).
2. In these studies, it is not clear how the outcome was assessed. It is assumed from the text that this was through the medical records, but this cannot be concluded with certainty.
3. The timepoint of determination of outcome is not mentioned.
4. Outcome is determined at 24 h, which is suboptimal for determining neurological outcome, and should preferably be longer.
5. Newcastle-Ottawa scale

As downloaded from <https://www.ohri.ca/programs/clinical_epidemiology/nosgen.pdf>.


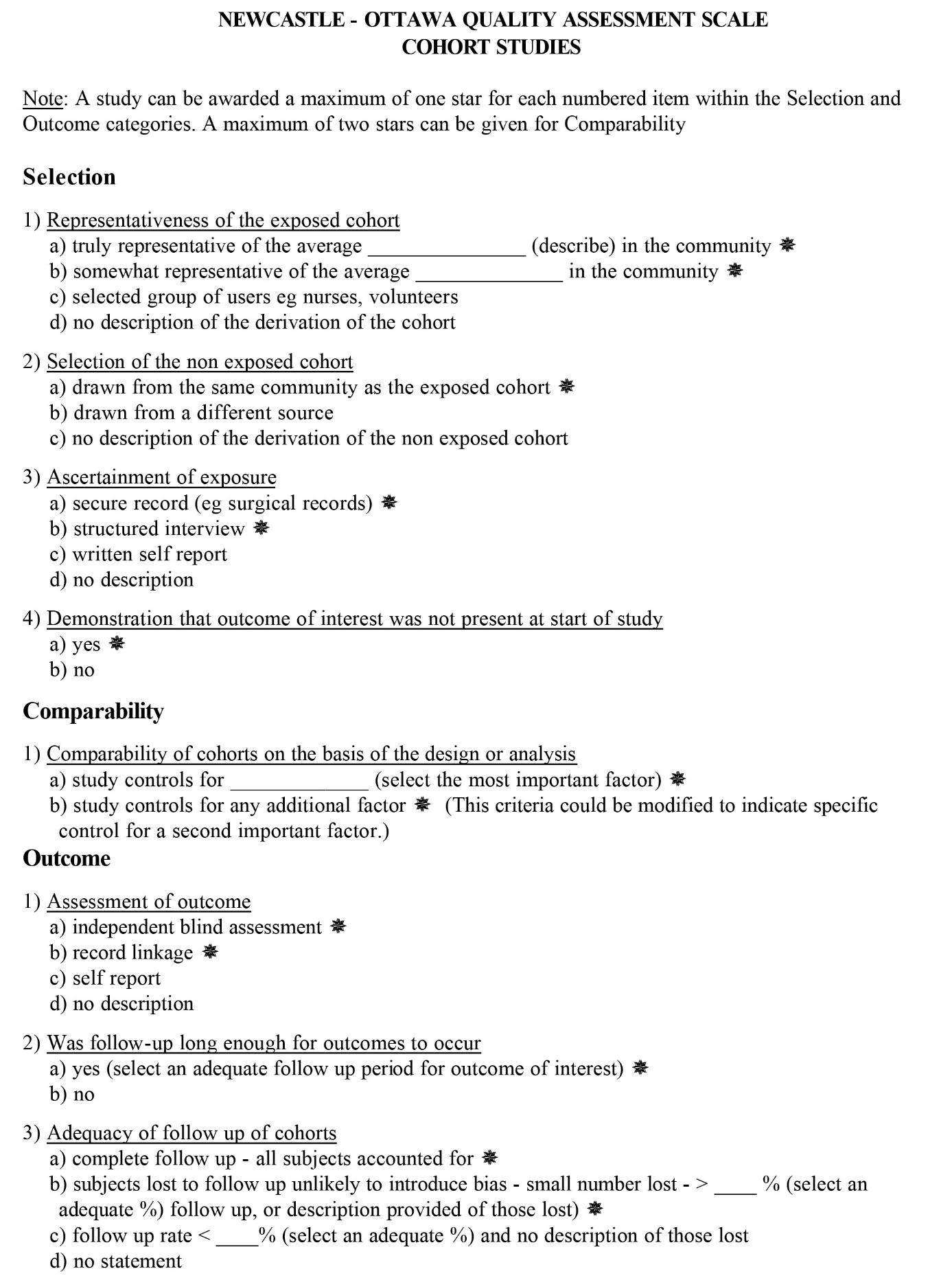


1. Additional study information

| Author | Cohort period | Causative procedures | Symptom severity | Timepoint of outcome determination |
| --- | --- | --- | --- | --- |
| *Studies with only group data* | | | | |
| Beevor | 1998-2014 | cardiac surgery: 24 (53%); central venous catheter: 8 (18%); trauma: 5 (11%); other surgery: 4 (8.9%); interventional radiology: 4 (8.9%) | cannot be reliably determined from the article; two patients were in circulatory arrest | hospital discharge |
| Blanc | 1980-1999 | central venous catheter: 52 (60%); extracorporeal circulation: 14 (16%); arteriography: 5 (5.8%); dialysis: 4 (4.7%); neurosurgery: 4 (4.7); others: 7 (8.1%) | cannot be reliably determined from the article | at least 2 months |
| *Studies with individual patient data* | | | | |
| Benson | 1987-1999 | A: 9 (47%); B: 2 (11%); E: 4 (21%); F: 3 (16%); G: 1 (5.3%) | 0: 2 (11%); 1: 4 (21%); 2: 8 (42%); 3: 2 (11%); 4: 3 (16%) | 1 month |
| Kol | > 1985 | E: 6 (100%) | 3: 6 (100%) | not mentioned |
| Massey | “9 year period” | A: 3 (21%); B: 1 (7.1%); C: 2 (14%); E: 5 (36%); H: 3 (21%) | 5: 14 (100%) | not mentioned |
| Muller | 2016-2020 | A: 2 (20%); B: 1 (10%); D: 1 (10%); F: 4 (40%); G: 1 (10%); H: 1 (10%) | 1: 4 (40%); 3: 6 (60%) | median 5 months |
| Murphy | 1970-1984 | A: 7 (50%); B: 1 (7,1%); C: 1 (7.1%); D: 1 (7.1%); E: 2 (14%); F: 2 (14%) | 1: 5 (36%); 2: 2 (14%); 3: 7 (50%) | not mentioned |
| Takahashi | 1970-1985 | A: 2 (11%); C: 7 (39%); D: 2 (11%); E: 7 (39%) | 5: 18 (100%) | not mentioned |
| Tekle | 1987-2010 | A: 15 (44%); B: 4 (12%); C: 2 (5.9%); E: 7 (21%); F: 2 (5.9%); G: 2 (5.9%); H: 1 (2.9%); I: 1 (2.9%) | 0: 2 (5.9%); 1: 11 (32%); 2: 10 (29%); 3: 8 (24%); 4: 3 (8.8%) | 24 h |
| Ziser | 1985-1998 | E: 17 (100%) | 5: 17 (100%) | hospital discharge |

In the two studies that did not report individual patient data, causative procedures and symptom severity could not be determined in the same way and to the same level of detail as in the remaining studies. For the studies that report individual patient data, the letters A-I in the third column and numbers 0-5 in the fourth column correspond to the letters and numbers in the table below.

| **Causative procedures categories**  A – venous / central venous gas entry B – aortic / carotid / cerebral gas entry C – coronary / cardiac catheterization D – peripheral arterial gas entry E – cardiac surgery F – lung procedures (including chest drain) G – pulmonary barotrauma (non-diving related) H – other I – unknown | **Symptom severity categories**  0 – nonspecific symptoms 1 – focal neurological symptoms 2 – altered mental status 3 – coma 4 – circulatory arrest 5 – unknown |
| --- | --- |
